# Knowledge, attitudes, and behavior of incarcerated people regarding COVID-19 and related vaccination: a survey in Italy

**DOI:** 10.1038/s41598-022-04919-3

**Published:** 2022-01-19

**Authors:** Gabriella Di Giuseppe, Concetta P. Pelullo, Raffaele Lanzano, Francesco Napolitano, Maria Pavia

**Affiliations:** Department of Experimental Medicine, University of Campania “Luigi Vanvitelli”, Via Luciano Armanni, 5, 80138 Naples, Italy

**Keywords:** Diseases, Infectious diseases

## Abstract

The cross-sectional study assessed knowledge, attitudes, and preventive practices toward COVID-19 disease of incarcerated people. A total of 685 subjects were surveyed. 94% were aware that respiratory droplets are involved in the transmission of COVID-19, and 77.2% that patients with chronic conditions are at risk of a more severe disease. Overall, 92.7% of respondents considered COVID-19 a more severe disease compared to influenza, and 85.4% believed that COVID-19 could cause serious consequences in their institution. Only 22.6% were self-confident about their ability to protect themselves from SARS-CoV-2 infection. This attitude was significantly higher in those who were involved in working activities in the institution, who did not report at least one common symptom compatible with COVID-19 in the previous 3 months, who did not show generalized anxiety symptoms, and did not need additional information. 63.9% of incarcerated people expressed willingness to receive COVID-19 vaccination. Older subjects, who knew that a COVID-19 vaccination is available, believed that COVID-19 is more serious than influenza, and were self-confident about their ability to protect themselves from SARS-CoV-2 infection, were significantly more willing to undergo COVID-19 vaccination. Public health response to COVID-19 in prisons should address vaccine hesitancy to increase vaccine confidence among incarcerated people.

## Introduction

Incarcerated people are a vulnerable population in respect to the risk of acquiring infectious diseases, including COVID-19, due to various conditions, such as overcrowding, confinement, poor hygiene, and reduced access to health care^1^. These conditions are to some extent shared with other institutionalized settings; moreover, the overall health of incarcerated subjects is on average poorer compared to the general population^2^, and non-communicable diseases, which have also been associated to more severe COVID-19 complications, are now the leading cause of mortality in prisons in many high-income countries^3,4^.

This underlying vulnerability has been further threated by the spread of COVID-19 throughout prisons, affecting incarcerated subjects and the personnel, and posing tremendous challenges to correctional facilities and public health authorities.

A recent systematic review investigating the management of highly contagious diseases outbreaks in prisons, including COVID-19, has highlighted that screening, contact tracing and isolation are the most applicable infection control strategies, whereas symptom screening can be ineffective as a consequence of fear of stigma, lack of trust in medical confidentiality, and willingness to avoid prolonged medical isolation^5^. The Authors conclude that up-to-date information about health risks, and prevention and control measures being implemented should be clearly communicated to incarcerated people and correctional staff.

As expected, the burden of COVID-19 has been very relevant in prisons all over the world, and outbreaks have involved both incarcerated people and staff. In the US the COVID-19 case rate for people in prison has been estimated to be 5.5 times higher than the US population case rate of 587 per 100,000, as well as the crude COVID-19 death rate (39 deaths per 100,000 incarcerated people), compared to the US population rate (29 deaths per 100,000)^6^, and 394,066 COVID-19 cases and 2555 deaths due to COVID-19 had been reported among incarcerated people in US by April 3, 2021^7^. In Italy, on April 2, 2020, the first prisoner died of COVID-19^8^, and 18 deaths have been reported among incarcerated subjects throughout the pandemic^9^. Data on COVID-19 cases among people in prison are reported weekly by the Ministry of Justice and the number of cases per 10,000 incarcerated people has always been higher than in the general population. The latest data available in Italy refer to December 6, 2021, reporting 196 COVID-19 positive out of 54,111 incarcerated people^10^.

Several investigations have evaluated the knowledge, perceptions, and preventive practices toward COVID-19 disease and the willingness to receive COVID-19 vaccination in different populations, such as adults^11–13^, healthcare workers (HCWs)^14,15^, and parents^16–18^. However, although incarcerated people represent one of the priority groups to be protected from the SARS-CoV-2^19^, very few information is available on their awareness, perceptions, and behaviours regarding the COVID-19 prevention, as well as on their self-confidence in protecting themselves from SARS-CoV-2 infection and intention to undergo COVID-19 vaccination^20,21^. Therefore, understanding the awareness and behaviour regarding COVID-19, and the willingness to receive the relative vaccination of people in prison and evaluating the determinants of these outcomes may help prison health authorities to develop effective preventive strategies in order to contain the spread of SARS-CoV-2 infection in this at-risk group.

The present study was carried out to explore knowledge, perceptions and preventive practices of incarcerated people toward COVID-19 disease, with specific attention to willingness to receive COVID-19 vaccination.

## Results

### Socio-demographic, detention, and anamnestic characteristics of the participants

Of the 850 subjects who were approached for the study, 165 refused to reply to the questionnaire because they did not consider the investigation useful to improve their health condition and 685 agreed to participate, with a participation rate of 80.6%. The main characteristics of the study population are reported in Table 1. The participants were between 18 and 78 years old (mean 42.4), almost all were Italian, only 25.8% had obtained a high school or university degree, 60% were married, 76.2% had sons/daughters, and slightly more than half (53.3%) were employed before detention. The vast majority (92.7%) lived in shared cells, for 41.4% this was the first episode of incarceration, and 28.6% reported a working activity in the prison. Moreover, 26.7% of the respondents were affected by chronic diseases, 62.3% reported at least one common symptom compatible with COVID-19 in the previous 3 months, and 86.7% had undergone a screening test with RT-PCR for SARS-CoV-2 detection.

**Table 1:** Selected socio-demographic and anamnestic characteristics and the associated willingness to receive COVID-19 vaccination (N = 865).

	Total		Willingness to receive COVID-19 vaccination^d^		
	N	%	N	%	
**Socio-demographic:**					
**Institution**					
Prison 1	248	36.2	107	44	
Prison 2	203	29.6	139	70.2	
Prison 3	234	34.2	182	79.5	
			χ^2^ = 69.05, 2 df, *p* < 0.001		
**Age**^**c**^	42.4 ± 11.93 (18–78)^a^		*t* test = -4.42, df = 666, *p* < 0.001		
**Nationality**^**c**^					
Italians	645	94.7	399	63.3	
Foreigners	36	5.3	26	72.2	
			χ^2^ = 1.16, 1 df, *p* = 0.280		
**Marital status**^**c**^					
Married/cohabitant	402	60.0	250	63.6	
Unmarried/widowed/separated/divorced	268	40.0	173	65.8	
			χ^2^ = 0.32, 1 df, *p* = 0.570		
**Sons/daughters**^**c**^					
Yes	508	76.2	319	64.3	
No	159	23.8	96	61.5	
			χ^2^ = 0.39, 1 df, *p* = 0.530		
**Education level**^**c**^					
High school or university degree	173	25.8	119	70	
Other	497	74.2	295	60.8	
			χ^2^ = 4.56, 1 df, *p* = 0.033		
**Occupation before detention**^**c**^					
Employed	360	53.3	237	67.1	
Unemployed	315	46.7	184	59.9	
			χ^2^ = 3.69, 1 df, *p* = 0.055		
**First detention**^**c**^					
Yes	273	41.4	163	60.8	
No	387	58.6	247	65.5	
			χ^2^ = 1.49, 1 df, *p* = 0.222		
**Working activity in the prison**					
Yes	196	28.6	133	69.6	
No	489	71.4	295	61.6	
			χ^2^ = 3.83, 1 df, *p* = 0.050		
**Type of cell**^**c**^					
Individual	45	7.3	38	86.4	
Shared	570	92.7	348	62.2	
			χ^2^ = 10.29, 1 df, *p* = 0.001		
**Anamnestic:**					
**At least one chronic disease**					
Yes	183	26.7	131	72.4	
No	502	73.3	297	60.7	
			χ^2^ = 7.76, 1 df, *p* = 0.005		
**At least one common symptom compatible with COVID-19 in the previous 3 months**^b^					
Yes	427	62.3	267	64	
No	258	37.7	161	63.6	
			χ^2^ = 0.01, 1 df, *p* = 0.918		
**Having generalized anxiety symptoms**					
Yes	354	51.7	214		61.8
No	331	48.3	214		66
			χ^2^ = 1.28, 1 df, *p* = 0.258		
**Having depression symptoms**					
Yes	310	45.3	194		64
No	375	54.7	234		63.8
			χ^2^ = 0.005, 1 df, *p* = 0.943		
**Having undergone a screening test with RT-PCR for SARS-CoV-2 detection**^**c**^					
Yes	569	86.7	352	63.2	
No	87	13.3	56	65.9	
			χ^2^ = 0.23, 1 df, *p* = 0.632		

^a^Mean ± standard deviation (range).

^b^Fever, cough, difficulty breathing, cold, sudden loss or decrease of smell, loss or alteration of taste, diarrhea, sore throat.

^c^Number for each item may not add up to total number of study population due to missing values. ^d^The frequency values of the respondents regarding the willingness to receive COVID-19 vaccination do not refer to the total number of the selected population due to the missing values.

### Knowledge about COVID-19 and related prevention

Overall, 94% of incarcerated people knew that respiratory droplets are involved in the transmission of COVID-19, 93.1% that COVID-19 can be transmitted by touching the mouth, nose and eyes with contaminated hands, and 77.2% that patients with chronic conditions are at risk of a more severe disease. Regarding prevention, 80.3% knew that a COVID-19 vaccine is available, and 96.6%, 95.6% and 95.2% mentioned hand washing, face masks use and physical distancing as effective infection control measures for COVID-19, respectively. Moreover, 19.6% knew the effective infection control measures for COVID-19 (Table 2).

**Table 2:** Knowledge about COVID-19 and the associated willingness to receive COVID-19 vaccination (N = 865).

Knowledge about COVID-19	Total		Willingness to receive COVID-19 vaccination^a^	
	N	%	N	%
**COVID-19 can be transmitted through sneezing and coughing**				
Yes	644	94	405	64.3
No	41	6	23	57.5
			χ^2^ = 0.75, 1 df, *p* = 0.386	
**COVID-19 can be transmitted by touching the mouth, nose and eyes with contaminated hands and not by mosquitoes bites**				
Yes	341	49.8	228	67.6
No	344	50.2	200	60.1
			χ^2^ = 4.19, 1 df, *p* = 0.041	
**Effectiveness of alcohol-based hand sanitizers against transmission**				
Yes	332	48.5	204	63
No	353	51.5	224	64.7
			χ^2^ = 0.23, 1 df, *p* = 0.632	
**Subjects with at least one chronic disease are at risk of severe complications of COVID-19**				
Yes	529	77.2	332	64
No	156	22.8	96	63.6
			χ^2^ = 0.008, 1 df, *p* = 0.929	
**COVID-19 vaccination is available**				
Yes	550	80.3	370	68.9
No	135	19.7	58	43.6
			χ^2^ = 29.55, 1 df, *p* < 0.001	
**Knowledge about effective infection control measures for COVID-19**				
Yes	134	19.6	74	56.5
No	551	80.4	354	65.7
			χ^2^ = 3.86, 1 df, *p* = 0.050	

^a^The frequency values of the respondents regarding the willingness to receive COVID-19 vaccination do not refer to the total number of the selected population due to the missing values.

### Attitudes about COVID-19 and related prevention

Severity of the disease was perceived by 92.7% of respondents who agreed that COVID-19 is more serious than influenza, by 85.4% who believed that this disease could cause serious consequences in their institution, by 82.3% who perceived to be at high risk of severe complications caused by COVID-19, and by 62.8% who believed that COVID-19 will continue to spread in Italy. Moreover, 38.7% considered themselves to be at risk of developing COVID-19, 32.5% believed that, even if necessary, they would prefer avoiding to go to the hospital due to the fear of contracting COVID-19, whereas only 22.6% were self-confident about their ability to protect themselves from SARS-CoV-2 infection (Table 3).

**Table 3:** Attitudes about COVID-19 and the associated willingness to receive COVID-19 vaccination (N = 865).

Attitudes about COVID-19	Total		Willingness to receive COVID-19 vaccination^b^	
	N	%	N	%
**Belief that COVID-19 is more serious than influenza**				
Agree	635	92.7	406	65.3
Uncertain/disagree	50	7.3	22	45.8
			χ^2^ = 7.3, 1 df, *p* = 0.007	
**Perception to be at high risk of severe complications caused by COVID-19**				
Agree	564	82.3	355	64.5
Uncertain/disagree	121	17.7	73	60.8
			χ^2^ = 0.59, 1 df, *p* = 0.443	
**Belief that, even if necessary, they would prefer avoiding to go to the hospital due to the fear of contracting COVID-19**				
Agree	223	32.5	128	59
Uncertain/disagree	462	67.5	300	66.2
			χ^2^ = 3.33, 1 df, *p* = 0.068	
**Perception to be at risk of developing COVID-19**				
Agree	265	38.7	164	63.6
Uncertain/disagree	420	61.3	264	64.1
			χ^2^ = 0.02, 1 df, *p* = 0.893	
**Belief that COVID-19 could cause serious consequences in their prison institution**				
Agree	585	85.4	366	63.9
Uncertain/disagree	100	14.6	62	63.9
			χ^2^ = 0.0001, 1 df, *p* = 0.993	
**Belief that COVID-19 will continue to spread in Italy**				
Agree	430	62.8	247	59.1
Uncertain/disagree	255	37.2	181	71.8
			χ^2^ = 11.05, 1 df, *p* = 0.001	
**Self-confidence about ability to protect oneself from SARS-CoV-2 infection**				
Agree	155	22.6	109	71.7
Uncertain/disagree	530	77.4	319	61.6
			χ^2^ = 5.22, 1 df, *p* = 0.022	

Sources of information about COVID-19	N	%	N	%
**Physicians**^**a**^				
Yes	114	16.9	76	69.7
No	561	83.1	347	62.9
			χ^2^ = 1.86, 1 df, *p* = 0.173	
**Media and newspaper**^**a**^				
Yes	638	94.5	401	64.2
No	37	5.5	22	61.1
			χ^2^ = 0.14, 1 df, *p* = 0.711	
**Family and friends**^**a**^				
Yes	236	35	162	71
No	439	65	261	60.3
			χ^2^ = 7.53, 1 df, *p* = 0.006	
**Prisoners involved in a prison education program**^**a**^				
Yes	34	5.2	22	68.7
No	623	94.8	389	63.7
			χ^2^ = 0.34, 1 df, *p* = 0.559	
**Need of additional information about COVID-19**				
Yes	506	75.6	315	63.8
No	163	24.4	104	64.6
			χ^2^ = 0.04, 1 df, *p* = 0.849	

^a^Number for each item may not add up to total number of study population due to missing values. ^b^The frequency values of the respondents regarding the willingness to receive COVID-19 vaccination do not refer to the total number of the selected population due to the missing values.

More than half (51.7%) and almost half (45.3%) were screened positive for symptoms of generalized anxiety and depression, respectively. Almost two thirds of the participants (63.9%) expressed willingness to receive COVID-19 vaccination and the most commonly reported reasons were the reduction of risk of infection (64.7%), the effectiveness (48.9%) and safety (48.2%) of the vaccine, that they believed themselves at high-risk of developing COVID-19 (47%), whereas only 19.2% reported that it had been recommended by a physician. For incarcerated who declared their unwillingness to receive a future COVID-19 vaccination, the main barriers were the concern about the safety (64.7%) and effectiveness (27.6%) of the vaccine, 25.4% believed it was not useful, and 22.4% reported that it was not recommended by a physician. The willingness to receive COVID-19 vaccination according to the socio-demographic and anamnestic characteristics, knowledge, and attitudes about COVID-19 of incarcerated is reported in Tables 1, 2 and 3.

### Behaviors related to COVID-19 prevention

Practice of protective behaviors was reported by 83.6% of participants who declared that they used face masks when they were seen by a doctor, or when they left the cell during working hours (84.7%) or yard time (80.3%). The majority of the respondents wash/disinfect their hands during yard time (81.9%), when they leave the cell during working hours (79.7%), and when they were seen by a doctor (71%). Moreover, 81.3% and 79.3% declared to practice hand washing before eating and after using the bathroom, respectively.

Almost two thirds of the participants (67%) reported to have modified their habits, in the 3 months preceding the survey, as a consequence of fear of contracting COVID-19, and the most commonly reported behaviors were avoiding close contacts (12.7%), gatherings (11.1%), socialization with other people in prison (10.5%), and practicing physical distancing (7%). Moreover, more than half (51.6%) expressed intention to avoid some behaviors in the next days for fear of contracting COVID-19, such as gatherings (11%), close contacts (9.9%), socialization with other incarcerated people (7.4%), and practice some preventive behaviors such as physical distancing (5.7%).

### Multivariate regression analysis

To have insight into the independent role of multiple determinants of the outcomes of interest, multivariate logistic regression models were built and the results are reported in Table 4. In the model performed to explore the association between the participants’ perception of risk of developing COVID-19, those who felt to be at high risk were significantly more likely to be affected by chronic diseases (OR = 2.68; 95% CI 1.73–4.16), to report at least one common symptom compatible with COVID-19 in the previous 3 months (OR = 1.55; 95% CI 1.03–2.35), to be older (OR = 1.06; 95% CI 1.04–1.08), to perceive to be at high risk of severe complications caused by COVID-19 (OR = 2.4; 95% CI 1.32–4.35), to believe that COVID-19 will cause serious consequences in their institution (OR = 3.12; 95% CI 1.59–6.12), to be less aware of the effective infection control measures for COVID-19 (OR = 0.56; 95% CI 0.34–0.92), and to believe that COVID-19 will continue to spread in Italy (OR = 1.86; 95% CI 1.21–2.86) (Model 1 in Table 4).

**Table 4:** Multivariate logistic regression analyses to characterize factors associated with the outcomes of interest.

	OR	SE	95% CI	*p*-value
**Model 1. Perceived risk of developing COVID-19**				
Log likelihood = − 330.14; χ^2^ = 190.43 (14 *df*); *p* < 0.0001				
Older	1.06	0.01	1.04–1.08	< 0.001
Being affected by chronic diseases	2.68	0.6	1.73–4.16	< 0.001
Reporting at least one common symptom compatible with COVID-19 in the previous 3 months	1.55	0.33	1.03–2.35	0.037
Poor knowledge about effective infection control measures for COVID-19	0.56	0.14	0.34–0.92	0.023
Believing that COVID-19 will cause serious consequences in their prison	3.12	1.07	1.59–6.12	0.001
Perceiving to be at high risk of severe complications caused by COVID-19	2.4	0.73	1.32–4.35	0.004
Believing that COVID-19 will continue to spread in Italy	1.86	0.41	1.21–2.86	0.005
*Institution*				
Prison 1	1^a^			
Prison 2	0.67	0.14	0.43–1.02	0.062
Married/cohabitant	1.37	0.3	0.89–2.1	0.152
Not having sons/daughters	0.67	0.18	0.39–1.14	0.142
Having had depression symptoms	1.55	0.38	0.96–2.51	0.075
Having had generalized anxiety symptoms	1.24	0.31	0.76–2.01	0.392
Not having received information about COVID-19 from family and friends	0.75	0.15	0.5–1.12	0.159
Need of additional information about COVID-19	1.42	0.34	0.88–2.27	0.149
**Model 2. Self-confidence about the ability to protect themselves from SARS-CoV-2 infection**				
Log likelihood = − 305.41; χ^2^ = 74.67 (9 *df*); *p* < 0.0001				
Not reporting at least one common symptom compatible with COVID-19 in the previous 3 months	0.45	0.01	0.3–0.69	< 0.001
*Institution*				
Prison 1	1.79	0.38	1.18–2.73	0.006
Prison 2	1^a^			
Working activity inside the prison	2.31	0.5	1.51–3.54	< 0.001
No generalized anxiety symptoms	0.61	0.13	0.4–0.92	0.019
No need of additional information about COVID-19	0.46	0.1	0.3–0.71	< 0.001
Foreigners	0.57	0.23	0.26–1.26	0.163
Not believing that COVID-19 will cause serious consequences in their prison	0.65	0.17	0.39–1.09	0.103
Poor knowledge about effective infection control measures for COVID-19	0.78	0.2	0.46–1.3	0.339
Having received information about COVID-19 from physicians	1.47	0.38	1.18–2.73	0.132
**Model 3. Willingness to receive COVID-19 vaccination**				
Log likelihood = − 343.85; χ^2^ = 126.81 (10 *df*); *p* < 0.0001				
Older	1.04	0.009	1.02–1.06	< 0.001
*Institution*				
Prison 1	1^a^			
Prison 2	2.71	0.62	1.73–4.25	< 0.001
Prison 3	4.46	1.07	2.78–7.14	< 0.001
Knowing that a COVID-19 vaccination is available	2.61	0.6	1.67–4.1	< 0.001
Believing that COVID-19 is more serious than influenza	2.21	0.84	1.05–4.66	0.037
Self-confidence about the ability to protect themselves from SARS-CoV-2 infection	1.65	0.4	1.03–2.64	0.038
High school or university degree	1.34	0.29	0.87–2.06	0.176
Being affected by chronic diseases	1.24	0.28	0.79–1.94	0.341
Not believing that COVID-19 will continue to spread in Italy	0.82	0.16	0.55–1.21	0.321
Having received information about COVID-19 from family and friends	1.34	0.27	0.9–2.01	0.150

^a^Reference category.

Self-confidence about the ability to protect themselves from SARS-CoV-2 infection, which was explored in Model 2, showed to be significantly higher in those who were involved in working activities in the institution (OR = 2.31; 95% CI 1.51–3.54), who did not report at least one common symptom compatible with COVID-19 in the previous 3 months (OR = 0.45; 95% CI 0.3–0.69), who did not show generalized anxiety symptoms (OR = 0.61; 95% CI 0.4–0.92), and did not express need of additional information about COVID-19 (OR = 0.46; 95% CI 0.3–0.71); moreover significant differences in self-confidence were revealed in the different investigated prisons (Model 2 in Table 4).

Finally, older subjects (OR = 1.04; 95% CI 1.02–1.06), who knew that a COVID-19 vaccination is available (OR = 2.61; 95% CI 1.67–4.1), believed that COVID-19 is more serious than influenza (OR = 2.21; 95% CI 1.05–4.66), and were self-confident about their ability to protect themselves from SARS-CoV-2 infection (OR = 1.65; 95% CI 1.03–2.64), were significantly more willing to undergo COVID-19 vaccination, and significant differences in this willingness were also detected in the different institutions (Model 3 in Table 4).

### Sources of information about COVID-19

The most frequently mentioned sources to acquire information about COVID-19 were media and newspapers (94.5%), followed by family and friends (35%), whereas only 16.9% reported physicians. Moreover, only 5.2% of participants had been involved in a prison education program and 75.6% wished to receive additional information about COVID-19 (Table 3).

## Discussion

As far as we know, this study is one of the few investigations that have explored in detail, in the context of the COVID-19 pandemic, the perspective of incarcerated people as regards to awareness and prevention of SARS-CoV-2 infection. The results have added knowledge on an understudied population providing useful insights to policymakers for the development of effective interventions aimed at contrasting the circulation of SARS-CoV-2 in prisons.

There are several relevant findings that warrant to be highlighted in respect to prevention of SARS-CoV-2 infection in people in prison.

First of all, after more than one year from the onset of the first cases of COVID-19 in Italy, the results of this study clearly show that knowledge about the main modes of transmission, such as the role of respiratory droplets as well as indirect transmission through contaminated hands, on severe consequences of COVID-19 for patients with underlying medical conditions, and on preventive measures, including vaccination, are widely spread in this population. These reassuring findings demonstrate that information on COVID-19 pandemic has reached also incarcerated subjects, and, although awareness of risks and of preventive measures does not directly imply the decision, ability or opportunity to implement protective behaviors, it may, however, provide the motivation and promote positive attitudes towards the adoption of preventive measures.

Moreover, the interpretation of results on attitudes has provided enlightening insights, since people in prison, expressing their concern about the possible consequences of COVID-19 for the potential for a huge spread in their institutions, as well as their specific risk of severe manifestations of COVID-19, have demonstrated a clear understanding of the seriousness of the pandemic for the threats to the safety of their institutional settings and, at the same time, for the implications related to the higher prevalence of medical conditions experienced by incarcerated people which are clearly perceived as a risk factor for severe complications of COVID-19. It is worth underlining, however, that this awareness is not coupled to self-confidence, since only one fifth declared to be confident in the ability to protect oneself from the SARS-CoV-2 infection. Therefore, since knowledge is satisfactory and attitudes show a conscious approach to the potential threats related to COVID-19, the lack of self-confidence appears to be related to perceived difficulties to protect themselves in that specific setting. The study did not directly investigate reasons of this lack of confidence, but it may be argued that it may be associated to the perception of overcrowding, since cells are shared by more than 90% of the participants, or, particularly in the first wave of the pandemic, by the lack or low availability of protective devices, such as face masks, hand disinfectants, etc.

These findings are coupled with a high level of anxiety and depression which were revealed to involve a consistent proportion of the studied population. Feelings of anxiety and depression have been frequently reported as a consequence of COVID-19 pandemic, even in subjects who did not directly experience the disease^22,23^, and this psychological distress may have been exacerbated by the condition of isolation related to incarceration. This is confirmed by the results of the model constructed to investigate determinants of self-confidence in the ability of incarcerated people to protect themselves from the SARS-CoV-2 infection, that showed that generalized feelings of anxiety were negatively associated to self-confidence, whereas a working activity, that may be considered a proxy of a more satisfactory psychological condition, was a predictor of self-confidence. Indeed, according to the Italian legislation, work in prison is one of the elements of the social rehabilitation during the detention period and, according to several requirements (condition of detention, unemployment gained during the detention, number of years spent in prison, etc.) incarcerated people are given the opportunity to carry out working activities, which include salary and social security guarantees.

Intention to undergo COVID-19 vaccination was expressed by 64% of the surveyed subjects. This finding is higher than that reported in the only study exploring willingness in people in prison in the US (45%)^24^, but lower than that expressed in the general population (84.1%), and in HCWs (80.7%) in the same area^25,26^, and in the general population in other areas such as in China, as reported by Wang et al. (82.6%)^11^ and by Chen et al. (83.3%)^12^, US (75%)^27^, and Canada (82.8%)^28^. This is expected since even in the general population lower willingness has been reported by disadvantaged groups and has been associated to less trust toward policies promoted by health institutions or the government^27,29^. It should be acknowledged, however, that the longer the time since the beginning of the pandemic, the higher the opportunity to increase one’s knowledge and awareness about the preventive measures, including vaccination. Therefore, comparing results from studies performed at different times, involving different populations, and using different methodologies should be done with caution.

It is also of note that, as recently reported by a comprehensive systematic review, data on coverage for all vaccinations in people in prison are scarce and heterogeneous, and do not include all relevant vaccines for this group; moreover, published literature indicate that incarcerated people are under-immunized, particularly against HBV, influenza, MMR, and pneumococci^30^. Reported reasons include specific characteristics of the people in prison and their fast turnover, and poor attention to prevention interventions in this disadvantaged group^30^. This is concerning, since COVID-19 has had a tremendous toll on both incarcerated people and personnel working in prisons, calling for actions aimed at urgent prioritization of COVID-19 vaccination in these settings. Reasons for considering COVID-19 vaccination a priority in prisons is also related to the high frequency of underlying chronic medical conditions revealed in people in prison, that are associated to COVID-19 more severe complications.

In this context, the analysis of determinants of willingness to undergo vaccination provides useful advice for promoting COVID-19 vaccination campaigns among people in prison, since it showed that younger incarcerated people, those who do not perceive the severity of the disease, and are not aware of the availability of the vaccine and feel uncomfortable with the preventive measures against COVID-19 should be the target of messages focused on the promotion of the adherence to COVID-19 vaccination as a crucial tool in the struggle against COVID-19 spread both in prisons and in the community.

Adherence to the main COVID-19 preventive measures was declared by the great majority of respondents, but there is still a proportion of more than 10% who reported not wearing face masks and not washing/disinfecting hands in various circumstances. This is alarming, since in the absence of vaccination, these measures, coupled with physical distancing, have proven to be fundamental for contrasting SARS-CoV-2 transmission. Regarding these results, it should be acknowledged that only 16.9% reported to have been informed on COVID-19 by a physician. Since in Italy healthcare provided to incarcerated people is managed by the National Health System, and it is well-known that HCWs are a very trusted source for health-related prevention behaviors, including vaccinations^31–34^, a more incisive role of HCW involved in healthcare of incarcerated people in the education and counselling about prevention of COVID-19 should be suggested. Indeed, among the reasons reported for willing to undergo COVID-19 vaccination, only 19.2% reported the recommendation by a physician, whereas 22.4% of those who would refuse vaccination declared they had been discouraged by a physician. This is unacceptable and demands a more thorough investigation involving directly HCW working in correctional facilities.

There are some potential limitations in the study that are worthy of emphasis and should be considered when interpreting the results. First, since this was a cross-sectional study, it can only provide associations and it does not enable establishing a causal and temporal relationship between the potential determinants and the different outcomes of interest. Moreover, incarcerated people were asked to respond to a hypothetical situation. Their indication that they will accept a COVID-19 vaccine may not correspond to true uptake rates; these will only be measurable when they will be actually offered a vaccine. Moreover, attitudes and perceptions might change quickly, and these results might not be reflective of current reasons for not intending to receive a COVID-19 vaccine. Third, incarcerated people were enrolled from three prisons of Southern Italy, hosting only male subjects and therefore the study examines a sub-set of the entire incarcerated population, potentially limiting the generalizability of the results to the wider population of incarcerated males and females in Italy. Surveys including women should be performed. It should also be acknowledged that the response rate was higher than the 64.2% and 68% reported in recent previous surveys conducted in prison^24,35^, therefore since the large majority of selected voluntarily participated, it is plausible to argue that the results of the survey would have not been substantially changed by the inclusion of those who were not recruited.

The study has demonstrated that the incarcerated people, a population with a high risk to acquire COVID-19, have acquainted an adequate knowledge about the main modes of transmission and the effective preventive measures, including vaccination. Moreover, they have shown a conscious approach to the potential threats related to COVID-19, whereas the lack of self-confidence in their ability to protect themselves from COVID-19 appears to be related to perceived difficulties associated to the specific setting. Finally, intention to undergo COVID-19 vaccine was not completely satisfactory. Therefore, public health response to COVID-19 in prisons should address vaccine hesitancy to increase vaccine confidence among people in prison given that early whole-institution vaccination can prevent outbreaks, ensure the basic rights of people in prisons, and protect staff and the wider community. Vaccine hesitancy is a very complex phenomenon and there is no definitive evidence available regarding specific effective interventions to address it, even if all the studies highlight the importance of understanding the specific concerns of various groups of hesitant individuals^36,37^. However, in the literature the impact of physicians’ recommendation on vaccination uptake among different at-risk groups has been well documented^38–41^. Therefore, prison HCWs should be more extensively involved in providing information and vaccine recommendation in order to increase the awareness of people in prison on the benefits of vaccination and to address their concerns.

## Methods

### Study design and setting

This cross-sectional investigation was conducted from March 9 to April 28, 2021, and it was part of a larger project developed by the University of Campania “Luigi Vanvitelli” and the Joint Operational Unit for “Health Protection at Prison Institutions”, to investigate several health-related issues in the incarcerated population. Three prisons in the geographic area of Campania region, in the South of Italy, hosting an average population of approximately 2125, 1164, and 954 adult incarcerated males, were included in the study. Each selected prison housed people awaiting trial and those who were definitively convicted, serving short or long sentences and consisted of sections that housed different groups according to low, medium or high security regimes.

A stratified sampling method was used to randomly select the study population. In particular, incarcerated people were stratified by their detention status. The strata were formed based on low, medium, or high security regime of incarcerated people and a proportional number of subjects were randomly selected from each group and invited to fill a self-administered questionnaire. Incarcerated women were excluded from sampling since a very small number of women are housed in women's enclosures of general prisons.

### Data collection

All directors of prisons were contacted through an invitation letter to present the study protocol and to obtain their approval to conduct the survey. Then, the selected subjects received an information sheet that explained the survey aims and procedures, that the participation was completely voluntary and anonymous, that privacy and confidentiality would be strictly protected, as no personal identifiers were included in the questionnaire. Moreover, a physician not involved in the care of incarcerated people, explained to them the purposes of the study, and a written informed consent was obtained before conducting the survey. The people in prison completed a self-administered questionnaire delivered in their prison cell. Those who were in special housing units or were unable to give informed consent for substantial cognitive impairment or were not able to read and comprehend Italian language were excluded.

### Survey questionnaire

The questionnaire was used to explore several topics, including: (1) characteristics of incarcerated people, and information on their current detention, such as age, marital status, number of sons/daughters, education level, employment status before and during detention, single or multiple episodes of incarceration, and living in individual or shared cells; (2) anamnestic characteristics, such as presence and type of underlying clinical conditions, having experienced the most common symptoms compatible with COVID-19 in the previous three months, and having undergone a screening test with RT-PCR for SARS-CoV-2 detection in the previous three months; (3) knowledge about COVID-19 (modes of transmission and preventive measures); (4) perceived severity and risk of developing the COVID-19, perceived ability to protect themselves from coronavirus, willingness or unwillingness to receive COVID-19 vaccine and related reasons, and level of anxiety and depression experienced during the pandemic period; (5) behavior regarding use of preventive measures, such as circumstances when masks were used and hands were washed/disinfected, eventual changes in their habits, in the three months preceding the survey, as a consequence of fear of contracting COVID-19, and related reason(s). Finally, (6) sources of and needs of information regarding COVID-19 were also explored.

The response choices for all knowledge questions were on a three-point Likert-type scale using "true", "false", "do not know" options. For the statements on perception of severity, risk of developing COVID-19, and for all attitudes, response options were also on a three-point Likert-type scale (1 = agree, 2 = uncertain, 3 = disagree). Depression was evaluated by using the “Patient Health Questionnaire 2” (PHQ-2) that has been shown to be effective in screening for depression and it comprised the first 2 questions of the “Patient Health Questionnaire 9” (PHQ-9), whereas anxiety was evaluated with the Generalized Anxiety Disorder 2-item (GAD-2), a validated test to perform screening for anxiety disorder^42,43^. Questions pertaining to willingness to receive the COVID-19 vaccine and those on behaviors were close ended with “yes” or “no” or multiple choices response format. A copy of the questionnaire is provided as additional file (Supplementary File S1).

Prior to the beginning of the survey, the questionnaire was pilot tested on 50 subjects to ensure correct interpretation, reliability and feasibility of the questions. The survey instrument was developed by research team based on items of questionnaires validated in previous investigations conducted by some of us regarding the knowledge, attitudes, and behavior about COVID-19 and other vaccinations in several at-risk groups^25,26,31–33^.

The study was approved by the Ethics Committees "Campania Centro" of the Local Health Unit Napoli 1 (protocol code: 297) and “Campania Nord” of the Local Health Unit Caserta (protocol code: 400). The research was conducted in accordance with relevant guidelines and regulations.

### Statistical analysis

The statistical Stata software (Version 15) was used to perform the analysis^44^. Descriptive analysis was used to explore the characteristics of the study population. Then, appropriate statistic tests (chi-square, Fisher’s exact and Student’s t-test) were conducted in bivariate analysis. Following, multivariate stepwise logistic regression analysis was performed to investigate predictors of perceived risk of developing COVID-19 (no = 0; yes = 1) (Model 1), of self-confidence in the ability to protect oneself from COVID-19 (no = 0; yes = 1) (Model 2), and of willingness to receive COVID-19 vaccination (no = 0; yes = 1) (Model 3). The independent variables that showed to be associated at the univariate analysis and were judged to potentially have influence to the investigated outcomes were included in the appropriate model. A detailed description of the independent variables included in each model and the related categories is reported in an additional file (Supplementary File S2).

## Supplementary Information

Supplementary Information 1.

Supplementary Information 2.

## Data Availability

The datasets generated during and/or analysed during the current study are available from the corresponding author on reasonable request.

## References

[1] Reinhart E, Chen DL (2020). Incarceration and its disseminations: COVID-19 pandemic lessons from Chicago's Cook County Jail. Health Aff. (Millwood)..

[2] Massoglia M, Pridemore WA (2015). Incarceration and health. Annu. Rev. Sociol..

[3] Herbert K, Plugge E, Foster C, Doll H (2012). Prevalence of risk factors for non-communicable diseases in prison populations worldwide: A systematic review. Lancet..

[4] Stürup-Toft S, O'Moore EJ, Plugge EH (2018). Looking behind the bars: Emerging health issues for people in prison. Br. Med. Bull..

[5] Beaudry, G., Zhong, S., Whiting, D., Javid, B., Frater, J. & Fazel, S. Managing outbreaks of highly contagious diseases in prisons: a systematic review. *BMJ Glob. Health.* **5,** e003201 (2020). 10.1136/bmjgh-2020-003201 PMC7670855 33199278

[6] Saloner B, Parish K, Ward JA, Di Laura G, Dolovich S (2020). COVID-19 cases and deaths in federal and state prisons. JAMA.

[7] Marquez N, Ward JA, Parish K, Saloner B, Dolovich S (2021). COVID-19 incidence and mortality in federal and state prisons compared with the US population, April 5, 2020, to April 3, 2021. JAMA.

[8] Cingolani M, Caraceni L, Cannovo N, Fedeli P (2021). COVID-19 incidence and mortality in federal and state prisons compared with the US population, April 5, 2020, to April 3, 2021. JAMA.

[9] Antigone. Associazione “per i diritti e le garanzie nel sistema penale”. Carcere e COVID-19. Italian. Available from: https://www.rapportoantigone.it/diciassettesimo-rapporto-sulle-condizioni-di-detenzione/covid-e-pandemia-in-italia/.

[10] Ministero della Giustizia. Monitoraggio COVID-19 negli Istituti Penitenziari. Italian. Available from: https://www.giustizia.it/giustizia/it/mg_2_27.page.

[11] Wang Z (2022). The willingness of Chinese adults to receive the COVID-19 vaccine and its associated factors at the early stage of the vaccination programme: A network analysis. J. Affect. Disord..

[12] Chen M (2021). An online survey of the attitude and willingness of Chinese adults to receive COVID-19 vaccination. Hum. Vaccin. Immunother..

[13] Yoda, T. & Katsuyama, H. Willingness to receive COVID-19 vaccination in Japan. *Vaccines (Basel)*. **9**, 48 (2021). 10.3390/vaccines9010048 PMC7828811 33466675

[14] Shaw J (2021). Assessment of US Healthcare personnel attitudes towards Coronavirus Disease 2019 (COVID-19) vaccination in a large University Healthcare System. Clin. Infect. Dis..

[15] Verger P (2021). Attitudes of healthcare workers towards COVID-19 vaccination: a survey in France and French-speaking parts of Belgium and Canada, 2020. Euro Surveill..

[16] Bagateli LE (2021). COVID-19 vaccine hesitancy among parents of children and adolescents living in Brazil. Vaccines (Basel).

[17] Yılmaz, M., Sahin, M.K. Parents' willingness and attitudes concerning the COVID-19 vaccine: A cross-sectional study. *Int. J. Clin. Pract.* **75**, e1436 (2021). 10.1111/ijcp.14364 PMC8236907 33998108

[18] Wang Q (2021). Vaccine Hesitancy: COVID-19 and Influenza vaccine willingness among parents in Wuxi, China-a cross-sectional study. Vaccines (Basel).

[19] Simpson PL, Guthrie J, Jones J, Butler T (2021). Identifying research priorities to improve the health of incarcerated populations: Results of citizens' juries in Australian prisons. Lancet Public Health..

[20] Berk J (2021). Initial SARS-CoV-2 vaccination uptake in a correctional setting: cross-sectional study. JMIRx Med..

[21] Pettus-Davis, C., Kennedy, S.C., Veeh, C.A. Incarcerated individuals' experiences of COVID-19 in the United States. Int. J. Prison Health. 10.1108/IJPH-11-2020-0094/full/html (2021). 10.1108/IJPH-11-2020-0094 33760428

[22] Delmastro M, Zamariola G (2020). Depressive symptoms in response to COVID-19 and lockdown: A cross-sectional study on the Italian population. Sci. Rep..

[23] Wang C (2020). Immediate psychological responses and associated factors during the initial stage of the 2019 Coronavirus Disease (COVID-19) epidemic among the general population in China. Int. J. Environ. Res. Public Health..

[24] Stern MF (2021). Willingness to receive a COVID-19 vaccination among incarcerated or detained persons in correctional and detention facilities—four states, September-December 2020. MMWR Morb. Mortal Wkly Rep..

[25] Di Giuseppe G (2021). Surveying willingness toward SARS-CoV-2 vaccination of healthcare workers in Italy. Expert Rev Vaccines..

[26] Di Giuseppe, G., Pelullo, C.P., Della Polla, G., Pavia, M. & Angelillo, I.F. Exploring the willingness to accept SARS-CoV-2 vaccine in a university population in Southern Italy, September to November 2020. *Vaccines (Basel).* **9,** 275 (2021). 10.3390/vaccines9030275 PMC8003195 33803730

[27] Kelly BJ (2021). Predictors of willingness to get a COVID-19 vaccine in the US. BMC Infect. Dis..

[28] Syan SK (2021). COVID-19 vaccine perceptions and differences by sex, age, and education in 1367 community adults in Ontario. Front. Public Health..

[29] Daly M, Robinson E (2021). Willingness to vaccinate against COVID-19 in the US: Representative longitudinal evidence from April to October 2020. Am. J. Prev. Med..

[30] Vicente-Alcalde N, Ruescas-Escolano E, Harboe ZB, Tuells J (2020). Vaccination coverage among prisoners: A systematic review. Int. J. Environ. Res. Public Health..

[31] Pelullo CP, Di Giuseppe G (2018). Vaccinations among Italian adolescents: Knowledge, attitude and behavior. Hum. Vaccin. Immunother..

[32] Pelullo CP, Napolitano F, Di Giuseppe G (2018). Meningococcal disease and vaccination: Knowledge and acceptability among adolescents in Italy. Hum. Vaccin. Immunother..

[33] Bertoldo G, Pesce A, Pepe A, Pelullo CP, Di Giuseppe G (2019). Seasonal influenza: Knowledge, attitude and vaccine uptake among adults with chronic conditions in Italy. PLoS ONE.

[34] Di Giuseppe G, Pelullo CP, Mitidieri M, Lioi G, Pavia M (2020). Cancer prevention: Knowledge, attitudes and lifestyle cancer-related behaviors among adolescents in Italy. Int. J. Environ. Res. Public Health..

[35] Moore A, Cox-Martin M, Dempsey AF, Berenbaum Szanton K, Binswanger IA (2019). HPV vaccination in correctional care: knowledge, attitudes, and barriers among incarcerated women. J Correct Health Care..

[36] Dubé E, Gagnon D, MacDonald NE (2015). Strategies intended to address vaccine hesitancy: Review of published reviews. Vaccine..

[37] Jarrett C (2015). Strategies for addressing vaccine hesitancy—A systematic review. Vaccine..

[38] Doornekamp, L., van Leeuwen, L., van Gorp, E., Voeten, H. & Goeijenbier, M. Determinants of vaccination uptake in risk populations: a comprehensive literature review. *Vaccines (Basel)*. **8**, 480 (2020). 10.3390/vaccines8030480 PMC7563537 32867126

[39] Napolitano F, Della Polla G, Angelillo IF (2019). Knowledge, attitudes, and behaviors of parents towards recommended adult vaccinations: An explanatory survey in the geographic area of Naples, Italy. Int. J. Environ. Res. Public Health..

[40] Wu S (2017). Factors associated with the uptake of seasonal influenza vaccination in older and younger adults: a large, population-based survey in Beijing. China. BMJ Open..

[41] Doherty M (2016). Vaccination of special populations: Protecting the vulnerable. Vaccine..

[42] Kroenke K, Spitzer RL, Williams JB, Löwe B (2009). An ultra-brief screening scale for anxiety and depression: the PHQ-4. Psychosomatics.

[43] Löwe B (2010). A 4-item measure of depression and anxiety: validation and standardization of the patient health questionnaire-4 (PHQ-4) in the General Population. J. Affect Disord..

[44] Stata Corporation. Stata Reference Manual Release 15.1. Stata Corporation; College Station: TX, USA (2017).

