# Supplementary material for: Knowledge, attitudes, and behavior of incarcerated people regarding COVID-19 and related vaccination: a survey in Italy
Source: Sci Rep. 2022 Jan 19;12:960. doi: 10.1038/s41598-022-04919-3 (PMC8770777; doi:10.1038/s41598-022-04919-3)
Supplement: Supplementary file 1 — Supplementary Information 1. [file 41598_2022_4919_MOESM1_ESM.docx]

**QUESTIONNAIRE**

**KNOWLEDGE, ATTITUDES, AND BEHAVIOR OF INCARCERATED PEOPLE REGARDING COVID-19 AND RELATED VACCINATION: A SURVEY IN ITALY**

**MARK WITH AN X OR, WHEN INDICATED, REPLY BRIEFLY**

**A. SOCIO-DEMOGRAPHIC CHARACTERISTICS**

*The questions in this section are aimed to acquire information on your socio-demographic characteristics*

**A.1.** Year of birth _____ **A.2.** Nationality ________________________

**A.3.** What is your present marital status? □ Unmarried □ Married/Cohabitant □ Separated □ Divorced □ Widowed

**A.4.** Do you have children (sons/daughters)? □ No □ Yes, how many children do you have? _______

**A.5.** What is the highest education leaving certificate, diploma or education degree you have obtained?

□ Primary school □ Middle school □ High school □ University degree □ Other __________

**A.6.** Before this detention, what was your work activity? _______________________________________

**A.7.** Is this your first detention?

□ Yes □ No, I have had other detentions and I have already spent in prison______ years, and _____months

**A.8.** Do you have a work activity in prison? □ No □ Yes **A.9.** Type of prison cell: □ Individual □ Shared

**B. ANAMNESTIC CHARACTERISTICS**

*The questions in this section are aimed to acquire information on your anamnestic characteristics*

**B.1.** Do you have any chronic disease? (e.g., diabetes, high blood pressure, etc.)?

□ No □ Yes, which one(more than one disease allowed) ________________________________________

**B.2.** Have you experienced at least one common symptom compatible with COVID-19 in the previous 3 months? (more than one response is allowed)

□ fever ≥ 37.5°C and chills □ cough □ respiratory difficulties □ cold □ sudden loss or decrease in smell □ sore throat □ loss or change in taste □ diarrhoea □ other _____________________________

**B.3.** Have you undergone a screening test for SARS-CoV-2 detection in the previous three months? □ No □ Yes

**C. KNOWLEDGE**

*The questions in this section are aimed at acquiring information about your knowledge regarding COVID-19*

**C.1.** For each statement answer “true”, “false”, or “I do not know”

|  | **True** | **False** | **I do not know** |
| --- | --- | --- | --- |
| **C.1.1.** COVID-19 can be transmitted by respiratory droplets, sneezing, or coughing | □ | □ | □ |
| **C.1.2.** COVID-19 can be transmitted by touching the mouth, nose and eyes with contaminated hands (not yet washed) | □ | □ | □ |
| **C.1.3.** COVID-19 can be transmitted by mosquito bites | □ | □ | □ |
| **C.1.4.** Alcohol-based hand sanitizers do not protect against transmission of COVID-19 | □ | □ | □ |
| **C.1.5.** Patients with chronic conditions are at risk of a more severe disease | □ | □ | □ |
| **C.1.6.** A COVID-19 vaccine is currently available | □ | □ | □ |

**C.2.** To reduce the risk of infection it is useful to:

|  | **True** | **False** | **I do not know** |
| --- | --- | --- | --- |
| **C.2.1.** Wash hands with soap and water | □ | □ | □ |
| **C.2.2.** Clean potentially contaminated surfaces with an alcohol-based disinfectant | □ | □ | □ |
| **C.2.3.** Have a physical distancing from people who sneeze or cough | □ | □ | □ |
| **C.2.4.** Have a physical distancing of at least one meter from other people | □ | □ | □ |
| **C.2.5.** Use a face mask | □ | □ | □ |
| **C.2.6.** Perform physical activity regularly | □ | □ | □ |
| **C.2.7.** Take herbal food supplements | □ | □ | □ |

Continue…

**D. ATTITUDES**

*The questions in this section are aimed at acquiring information about your attitudes regarding COVID-19*

**D.1.** For each statement answer whether you agree, are uncertain, or disagree

|  | **Agree** | **Uncertain** | **Disagree** |
| --- | --- | --- | --- |
| **D.1.1.** COVID-19 is more serious than influenza | □ | □ | □ |
| **D.1.2.** I am at high risk of severe complications caused by COVID-19 | □ | □ | □ |
| **D.1.3.** Even if necessary, I would prefer avoiding to go to the hospital due to the fear of contracting COVID-19 | □ | □ | □ |
| **D.1.4.** I am more at risk of developing COVID-19 than other people | □ | □ | □ |
| **D.1.5.** COVID-19 could cause serious consequences in my prison institution | □ | □ | □ |
| **D.1.6.** COVID-19 will continue to spread in Italy | □ | □ | □ |
| **D.1.7.** I am confident about my ability to protect myself from SARS-CoV-2 infection | □ | □ | □ |

**D.2**. In the last two weeks, how often have you been bothered by any of the following problems due to the fear of contracting COVID-19?

**D.2.1.** Feeling nervous, anxious, or on edge

□ Not at all □ Several days □ More than half the days □ Nearly everyday

**D.2.2.** Not being able to control the worrying

□ Not at all □ Several days □ More than half the days □ Nearly everyday

**D.2.3.** Feeling down, depressed, or hopeless

□ Not at all □ Several days □ More than half the days □ Nearly everyday

**D.2.4.** Little interest or pleasure in doing things that I used to enjoy

□ Not at all □ Several days □ More than half the days □ Nearly everyday

**E. BEHAVIOURS**

*The questions in this section are aimed at exploring your behaviors regarding COVID-19*

**E.1.** In the last 3 month have you modified your habits in the last 3 months as a consequence of fear of contracting COVID-19?

□ No □ Yes, please specify __________________________________

**E.2.** In the next day do you expect to avoid any behaviors for fear of contracting COVID-19?

□ No □ Yes, please specify_____________________________________

**E.3.** In the last 3 months have you used a face mask when: (more than one response is allowed)

□ You were seen by a doctor?

□ You left the cell during working hours?

□ You left the cell during yard time?

□ I have never used the mask  □ Other ________________________

**E.4.** In the previous three months have you washed and/or disinfected your hands when (more than one response is allowed)

□ You were seen by a doctor?

□ You left the cell during working hours?

□ You left the cell during yard time?

□ After using the bathroom?

□ Before eating?

□ I have never disinfected my hands

□ Other ________________________

**E.5.** Would you get vaccinated against COVID-19?

□ No □ Yes ↓ ↓

| **Reasons** (more than one reason is allowed) | **Reasons** (more than one reason is allowed) |
| --- | --- |
| □ Vaccine is not useful | □ Vaccine reduces the risk of infection |
| □ Vaccine is not safe | □ Vaccine is safe |
| □ Vaccine is not effective | □ Vaccine is effective |
| □ Vaccine against COVID-19 is not recommended by physicians | □ Vaccine against COVID-19 is recommended by physicians |
| □ I do not feel at-risk | □ I am at high-risk of developing COVID-19 |
| □ Other, please specify ________________________ | □ Other, please specify __________________________ |

**F. INFORMATION**

*The questions in this section are aimed to evaluate the sources from which you acquire information on COVID-19 vaccination*

**F.1.** From which of the following sources do you receive information about COVID-19 vaccination? (more than one source is allowed)

□ None □ Physicians □ Media/newspapers □ Friends/Family □ Other, please specify ­___________

**F.2.** Have you been involved in a prison education program about COVID-19? □ No □ Yes

**F.3.** Do you feel you need additional information on COVID-19? □ No □ Yes

**Thank you for answering the questionnaire!**
