# Supplementary material for: Knowledge, attitudes, and behavior of incarcerated people regarding COVID-19 and related vaccination: a survey in Italy
Source: Sci Rep. 2022 Jan 19;12:960. doi: 10.1038/s41598-022-04919-3 (PMC8770777; doi:10.1038/s41598-022-04919-3)
Supplement: Supplementary file 2 — Supplementary Information 2. [file 41598_2022_4919_MOESM2_ESM.docx]

**Variables included in the logistic regression models with related categories**

**Model 1.** Perceived risk of developing COVID-19

| **Independent variables** | **Code** |
| --- | --- |
| Institution | Prison 1=1 |
|  | Prison 2=2 |
|  | Prison 3=3 |
| Age | Years (continuos) |
| Nationality | Foreigners=0 |
|  | Italians=1 |
| Marital status | Unmarried/widowed/separated/divorced=0 |
|  | Married/cohabitant=1 |
| Sons/daughters | No=0 |
|  | Yes=1 |
| Education level | Other=0 |
|  | High school or university degree=1 |
| Occupation before detention | Unemployed=0 |
|  | Employed=1 |
| First detention | No=0 |
|  | Yes=1 |
| Working activity in the prison | No=0 |
|  | Yes=1 |
| Type of cell | Individual= |
|  | Shared=1 |
| At least one chronic disease | No=0 |
|  | Yes=1 |
| At least one common symptom compatible with COVID-19 in the previous 3 months | No=0 |
|  | Yes=1 |
| Having generalized anxiety symptoms | No=0 |
|  | Yes=1 |
| Having depression symptoms | No=0 |
|  | Yes=1 |
| Having undergone a screening test with RT-PCR for SARS-CoV-2 detection | No=0 |
|  | Yes=1 |
| Knowledge that COVID-19 can be transmitted through sneezing and coughing | No=0 |
|  | Yes=1 |
| Knowledge about COVID-19 transmission | No=0 |
|  | Yes=1 |
| Effectiveness of alcohol-based hand sanitizers against transmission | No=0 |
|  | Yes=1 |
| Knowledge that subjects with at least one chronic disease are at risk of severe complications of COVID-19 | No=0 |
|  | Yes=1 |
| Knowledge that COVID-19 vaccination is available | No=0 |
|  | Yes=1 |
| Knowledge about effective infection control measures for COVID-19 | No=0 |
|  | Yes=1 |
| Belief that COVID-19 is more serious than influenza | Uncertain/Disagree=0 |
|  | Agree=1 |
| Perception to be at high risk of severe complications caused by COVID-19 | Uncertain/Disagree=0 |
|  | Agree=1 |
| Belief that, even if necessary, they would prefer avoiding to go to the hospital due to the fear of contracting COVID-19 | Uncertain/Disagree=0 |
|  | Agree=1 |
| Belief that COVID-19 could cause serious consequences in their prison institution | Uncertain/Disagree=0 |
|  | Agree=1 |
| Belief that COVID-19 will continue to spread in Italy | Uncertain/Disagree=0 |
|  | Agree=1 |
| Self-confidence about ability to protect oneself from SARS-CoV-2 infection | Uncertain/Disagree=0 |
|  | Agree=1 |
| Physicians as source of information | No=0 |
|  | Yes=1 |
| Media and newspaper as source of information | No=0 |
|  | Yes=1 |
| Family and friends as source of information | No=0 |
|  | Yes=1 |
| Prisoners involved in a prison education program | No=0 |
|  | Yes=1 |
| Need of additional information about COVID-19 | No=0 |
|  | Yes=1 |

**Model 2.** Self-confidence about the ability to protect themselves from SARS-CoV-2 infection

| **Independent variables** | **Code** |
| --- | --- |
| Institution | Prison 1=1 |
|  | Prison 2=2 |
|  | Prison 3=3 |
| Age | Years (continuos) |
| Nationality | Foreigners=0 |
|  | Italians=1 |
| Marital status | Unmarried/widowed/separated/divorced=0 |
|  | Married/cohabitant=1 |
| Sons/daughters | No=0 |
|  | Yes=1 |
| Education level | Other=0 |
|  | High school or university degree=1 |
| Occupation before detention | Unemployed=0 |
|  | Employed=1 |
| First detention | No=0 |
|  | Yes=1 |
| Working activity in the prison | No=0 |
|  | Yes=1 |
| Type of cell | Individual= |
|  | Shared=1 |
| At least one chronic disease | No=0 |
|  | Yes=1 |
| At least one common symptom compatible with COVID-19 in the previous 3 months | No=0 |
|  | Yes=1 |
| Having generalized anxiety symptoms | No=0 |
|  | Yes=1 |
| Having depression symptoms | No=0 |
|  | Yes=1 |
| Having undergone a screening test with RT-PCR for SARS-CoV-2 detection | No=0 |
|  | Yes=1 |
| Knowledge that COVID-19 can be transmitted through sneezing and coughing | No=0 |
|  | Yes=1 |
| Knowledge about COVID-19 transmission | No=0 |
|  | Yes=1 |
| Not usefulness of alcohol-based hand sanitizers against transmission | No=0 |
|  | Yes=1 |
| Knowledge that subjects with at least one chronic disease are at risk of severe complications of COVID-19 | No=0 |
|  | Yes=1 |
| Knowledge that COVID-19 vaccination is available | No=0 |
|  | Yes=1 |
| Knowledge about effective infection control measures for COVID-19 | No=0 |
|  | Yes=1 |
| Belief that COVID-19 is more serious than influenza | Uncertain/Disagree=0 |
|  | Agree=1 |
| Perception to be at high risk of severe complications caused by COVID-19 | Uncertain/Disagree=0 |
|  | Agree=1 |
| Belief that, even if necessary, they would prefer avoiding to go to the hospital due to the fear of contracting COVID-19 | Uncertain/Disagree=0 |
|  | Agree=1 |
| Perception to be at risk of developing COVID-19 | Uncertain/Disagree=0 |
|  | Agree=1 |
| Belief that COVID-19 could cause serious consequences in their prison institution | Uncertain/Disagree=0 |
|  | Agree=1 |
| Belief that COVID-19 will continue to spread in Italy | Uncertain/Disagree=0 |
|  | Agree=1 |
| Physicians as source of information | No=0 |
|  | Yes=1 |
| Media and newspaper as source of information | No=0 |
|  | Yes=1 |
| Family and friends as source of information | No=0 |
|  | Yes=1 |
| Prisoners involved in a prison education program | No=0 |
|  | Yes=1 |
| Need of additional information about COVID-19 | No=0 |
|  | Yes=1 |

**Model 3.** Willingness to receive COVID-19 vaccination

| **Independent variables** | **Code** |
| --- | --- |
| Institution | Prison 1=1 |
|  | Prison 2=2 |
|  | Prison 3=3 |
| Age | Years (continuos) |
| Nationality | Foreigners=0 |
|  | Italians=1 |
| Marital status | Unmarried/widowed/separated/divorced=0 |
|  | Married/cohabitant=1 |
| Sons/daughters | No=0 |
|  | Yes=1 |
| Education level | Other=0 |
|  | High school or university degree=1 |
| Occupation before detention | Unemployed=0 |
|  | Employed=1 |
| First detention | No=0 |
|  | Yes=1 |
| Working activity in the prison | No=0 |
|  | Yes=1 |
| Type of cell | Individual= |
|  | Shared=1 |
| At least one chronic disease | No=0 |
|  | Yes=1 |
| At least one common symptom compatible with COVID-19 in the previous 3 months | No=0 |
|  | Yes=1 |
| Having generalized anxiety symptoms | No=0 |
|  | Yes=1 |
| Having depression symptoms | No=0 |
|  | Yes=1 |
| Having undergone a screening test with RT-PCR for SARS-CoV-2 detection | No=0 |
|  | Yes=1 |
| Knowledge that COVID-19 can be transmitted through sneezing and coughing | No=0 |
|  | Yes=1 |
| Knowledge about COVID-19 transmission | No=0 |
|  | Yes=1 |
| Not usefulness of alcohol-based hand sanitizers against transmission | No=0 |
|  | Yes=1 |
| Knowledge that subjects with at least one chronic disease are at risk of severe complications of COVID-19 | No=0 |
|  | Yes=1 |
| Knowledge that COVID-19 vaccination is available | No=0 |
|  | Yes=1 |
| Knowledge about effective infection control measures for COVID-19 | No=0 |
|  | Yes=1 |
| Belief that COVID-19 is more serious than influenza | Uncertain/Disagree=0 |
|  | Agree=1 |
| Perception to be at high risk of severe complications caused by COVID-19 | Uncertain/Disagree=0 |
|  | Agree=1 |
| Belief that, even if necessary, they would prefer avoiding to go to the hospital due to the fear of contracting COVID-19 | Uncertain/Disagree=0 |
|  | Agree=1 |
| Perception to be at risk of developing COVID-19 | Uncertain/Disagree=0 |
|  | Agree=1 |
| Belief that COVID-19 could cause serious consequences in their prison institution | Uncertain/Disagree=0 |
|  | Agree=1 |
| Belief that COVID-19 will continue to spread in Italy | Uncertain/Disagree=0 |
|  | Agree=1 |
| Self-confidence about ability to protect oneself from SARS-CoV-2 infection | Uncertain/Disagree=0 |
|  | Agree=1 |
| Physicians as source of information | No=0 |
|  | Yes=1 |
| Media and newspaper as source of information | No=0 |
|  | Yes=1 |
| Family and friends as source of information | No=0 |
|  | Yes=1 |
| Prisoners involved in a prison education program | No=0 |
|  | Yes=1 |
| Need of additional information about COVID-19 | No=0 |
|  | Yes=1 |
